# Transverse Maxillary Correction: Leaf Expander vs. Rapid Maxillary Expansion Appliances—A Systematic Review and Meta-Analysis

**DOI:** 10.3390/children13030396

**Published:** 2026-03-12

**Authors:** Elena Caramaschi, Alessio Verdecchia, Maurizio Ledda, Claudia Dettori, Teresa Cobo, Alin Marian Iacob, Enrico Spinas

**Affiliations:** 1 Department of Surgical Sciences, Postgraduate School in Orthodontics, University of Cagliari, 09124 Cagliari, Italy; caramaschi.elena@gmail.com (E.C.); maurizioledda93@gmail.com (M.L.); 2 Orthodontics Division, Instituto Asturiano de Odontología, Universidad de Oviedo, 33006 Oviedo, Spain; dracobo@iaodontologia.com (T.C.); alini63@yahoo.com (A.M.I.); 3 Department of Surgical Sciences, School of Dental Medicine, University of Cagliari, 09124 Cagliari, Italy; claudia.dettori@gmail.com

**Keywords:** Leaf Expander, slow maxillary expansion, SME, rapid maxillary expansion, RME, Haas, Hyrax, maxillary transverse deficiency

## Abstract

**Background/Objectives:** Transverse maxillary deficiency in growing patients can be treated using rapid maxillary expansion (RME) or slow maxillary expansion (SME) with spring-based appliances, such as the Leaf Expander (LE), but their comparative dentoskeletal effects remain debated. This study evaluated the transverse dentoskeletal outcomes of LE-based SME versus conventional RME. **Methods:** A systematic review was conducted in accordance with PRISMA 2020 guidelines and registered in PROSPERO. Electronic searches were performed in PubMed, Scopus, Embase, Web of Science, and Cochrane Library up to 9 January 2026. Randomized controlled trials (RCTs) comparing LE-based SME and RME in skeletally immature patients were included. Primary outcomes were transverse maxillary change; secondary outcomes included dentoalveolar side effects. Risk of bias was assessed using the RoB 2 tool, and certainty of evidence was evaluated using the GRADE framework. When possible, a meta-analysis was performed using standardized mean differences and a random-effects model. **Results:** Four RCTs met the inclusion criteria. Both SME and RME achieved significant transverse expansion. Meta-analysis showed no statistically significant differences between protocols for inter-canine distance, inter-second deciduous molar distance, inter-first permanent molar distance, or basal maxillary width. Intergroup differences varied by anatomical site and measurement method: RME showed greater anterior dental and skeletal transverse gains, whereas SME achieved comparable intermolar expansion with greater molar distorotation. Three-dimensional analyses indicated similar morphological enlargement. Risk of bias ranged from low to high; the certainty of evidence was low to very low for most transverse parameters and moderate only for molar distorotation. **Conclusions:** Both LE-based SME and RME effectively correct transverse maxillary deficiency. Quantitative synthesis showed comparable overall transverse expansion, with differences mainly related to the distribution and biomechanical pattern of dentoskeletal effects rather than the absolute amount of expansion achieved. Appliance selection should be guided by biomechanical features and individual treatment objectives. Further high-quality RCTs with standardized three-dimensional protocols and longer follow-up are needed.

## 1. Introduction

Transverse maxillary deficiency is a common skeletal discrepancy in orthodontic practice and is frequently associated with posterior crossbite. It represents a clinically relevant condition in growing patients [1,2,3,4], as untreated transverse constriction may lead to occlusal disharmony, dental crowding, mandibular functional shifts, and altered craniofacial development [5,6,7,8]. For this reason, early diagnosis and correction of transverse maxillary discrepancies are recommended during the stages of active growth [9].

Maxillary expansion is the treatment of choice in skeletally immature patients, as it takes advantage of the relative patency of the midpalatal and circummaxillary sutures before pubertal maturation [10,11]. Histological and biomechanical evidence shows that transverse expansion produces both skeletal and dentoalveolar changes, whose relative contribution depends on age, appliance design, anchorage, and activation protocol [10,12,13,14,15].

Expansion protocols are broadly classified as rapid maxillary expansion (RME) and slow maxillary expansion (SME). RME and SME differ not only in force magnitude but also in activation modality and biomechanical response. RME typically uses tooth- or tissue-borne appliances activated through daily screw turns (commonly 0.25 mm per activation, one or two turns per day), generating heavy, intermittent forces over a short period and producing immediate midpalatal suture separation with rapid skeletal displacement [10,16,17]. Its effectiveness in increasing transverse maxillary dimensions and resolving posterior crossbite, including orthopedic effects at the circummaxillary sutures, is well documented [10,13,18]. However, high force levels have been associated with pain, soft tissue inflammation, buccal tipping of anchor teeth, periodontal side effects, and limited control of dentoalveolar compensation, particularly when permanent teeth are used for anchorage [14,19,20].

In contrast, SME appliances generally rely on elastic deformation of spring components, which may be fabricated from stainless steel, cobalt-chromium, or nickel-titanium alloys, to deliver sustained low magnitude forces over an extended activation period, promoting gradual transverse expansion through progressive sutural remodeling rather than abrupt mechanical separation [21,22]. The Leaf Expander^®^ (LE) represents a specific form of SME, as it incorporates preactivated nickel–titanium leaf springs that release calibrated, constant forces of predetermined intensity over time without repeated manual screw activations. This controlled force delivery reduces activation variability and eliminates the need for patient compliance [21]. Biomechanically, its mechanism induces gradual sutural remodeling through sustained low-magnitude force application, consistent with the biological principles of slow maxillary expansion and progressive dentoskeletal adaptation.

A major topic of debate in the literature concerns the relative dentoskeletal effects of the two expansion protocols, as although both RME and SME are effective in correcting transverse maxillary deficiency [23], the magnitude and distribution of skeletal versus dentoalveolar expansion remain controversial.

To date, despite the increasing number of randomized controlled trials (RCTs) comparing LE-based SME and conventional RME in growing patients, no systematic review has comprehensively synthesized the available evidence to clarify their relative effectiveness in transverse maxillary correction. A critical appraisal and systematic synthesis of RCTs is therefore needed; accordingly, this systematic review aimed to compare the effects of LE-based SME and conventional RME in growing patients, with a specific focus on transverse dentoskeletal outcomes and related secondary effects, in order to inform evidence-based treatment selection.

## 2. Materials and Methods

### 2.1. Protocol Registration

This systematic review was conducted in accordance with the Preferred Reporting Items for Systematic Reviews and Meta-analysis (PRISMA) 2020 guidelines [24] and prospectively registered in the International Prospective Register of Systematic Reviews (PROSPERO) (CRD420251234705). The full review protocol was written in advance and is publicly available at https://www.crd.york.ac.uk/PROSPERO/view/CRD420251234705 (accessed on 2 January 2026).

### 2.2. Study Question and PICO Framework

This systematic review was designed to address the following focused question: In skeletally immature patients with maxillary transverse deficiency, does SME performed with spring-based LE devices, compared with RME using conventional Hyrax- or Haas-type expanders, result in different amounts of maxillary transverse expansion, and is there evidence of a distinct skeletal versus dentoalveolar contribution to the observed transverse changes?

The review question was formulated according to the PICO framework as follows:

• Population: skeletally immature patients with maxillary transverse deficiency.
• Intervention: SME protocols using spring-based LE devices.
• Comparison: RME using rapid activation protocols with conventional Hyrax- or Haas-type expanders.
• Outcomes: the primary outcome was transverse maxillary change, evaluated through skeletal and dental transverse measurements (e.g., inter-permanent incisive width, inter-deciduous canine width, inter-second primary molar width, inter-first permanent molar width, midpalatal suture opening, palatal surface changes, and basal maxillary width); the secondary outcome was dentoalveolar effects, such as molar inclination/tipping and upper first molar distorotation.

### 2.3. Information Sources

PubMed (MEDLINE), Embase, Scopus, Web of Science, and the Cochrane Library were systematically searched from inception to 9 January 2026. No language filters were applied during the electronic search. The reference lists of all included studies were manually screened to identify additional eligible trials.

### 2.4. Search Strategy

Electronic searches were performed independently by two researchers (E.C., A.V.). The search strategy combined MeSH terms and free-text keywords related to maxillary transverse deficiency, maxillary expansion, LE, and rapid maxillary expansion. The complete search strategy is provided in Table S1 in the Supplementary Materials.

### 2.5. Eligibility Criteria

Only RCTs comparing SME with spring-based LE and RME with conventional tooth-borne appliances in skeletally immature patients were included. Detailed inclusion and exclusion criteria are reported in Table 1.

### 2.6. Study Selection

Duplicate records were removed using Zotero and manually verified for accuracy. Titles and abstracts of all identified records were independently screened by two operators (E.C., A.V.). Full-text articles were retrieved for studies that met the inclusion criteria or when eligibility could not be determined based on the title and/or abstract alone. Any disagreements between reviewers were resolved through discussion and consensus; when disagreement persisted, a third operator (E.S.) was consulted. When necessary, study authors were contacted to clarify missing or unclear information. Cohen’s kappa coefficient [25] for agreement between the reviewers was 0.89.

### 2.7. Data Collection

Data extraction was independently performed by two researchers (E.C., A.V.) using a standardized data extraction form. For each included study, the following information was extracted:

• Study characteristics.
• Patient characteristics (age, gender, cervical vertebral maturation, type of malocclusion, and stage of dentition).
• Diagnostic criteria for maxillary transverse deficiency (clinical examination, dental casts, and/or radiographic or 3D imaging assessments).
• Appliance type.
• Activation protocol.
• Treatment duration.
• Primary outcome.
• Secondary outcome.

When studies reported more than one measurement or assessment time point, data obtained at the completion of the active expansion phase were preferentially considered. Differences in data extraction between researchers (E.C., A.V.) were addressed through discussion and agreement and, when needed, by involving a third reviewer (E.S.). In cases of missing or ambiguous information, the corresponding authors were contacted for clarification.

### 2.8. Risk of Bias Assessment in Included Studies

The quality of the included RCTs was independently assessed by two researchers (E.C., A.V.) using the Cochrane Risk of Bias tool for randomized trials (RoB 2.0) [26].

The following biases were investigated for each included study:

• Bias arising from the randomization process.
• Bias due to deviations from the intended intervention.
• Bias due to missing outcome data.
• Bias in the measurement of the outcome.
• Bias in the selection of the reported result.
• Each domain was judged as “low risk of bias”, “some concerns”, or “high risk of bias”, according to the algorithm. Disagreements between researchers (E.C., A.V.) were resolved through discussion and consensus, and when necessary, by consultation with a third reviewer.

### 2.9. Certainty of Evidence

The certainty of evidence across outcomes was determined using the Grading of Recommendations Assessment, Development and Evaluation (GRADE) framework [27]. Certainty was subsequently downgraded in the GRADE domains of risk of bias, inconsistency, indirectness, imprecision, and publication bias, where relevant. The overall certainty of evidence for each outcome was classified as high, moderate, low, or very low.

### 2.10. Meta-Analysis

A meta-analysis was conducted to quantitatively synthesize the results of the included studies and compare the transverse dentoskeletal effects of SME with nickel–titanium LE and RME. The outcomes analyzed included transverse skeletal and dental measurements, such as inter-canine distance (IDC), inter-second deciduous molar distance (ISDM), inter-first permanent molar distance (IFPM), posterior skeletal opening measurements, and basal maxillary width (BMW). Because the included studies reported these parameters using different measurement scales and anatomical reference points, standardized mean differences (SMDs) were calculated to standardize the results across studies. When more than one measurement corresponding to the same parameter was reported within a study, these values were considered independent subgroups in the analysis. When the results were stratified according to diagnostic subgroups, averaged values were calculated to obtain a single estimate comparable with the other studies. Pooled SMD values between SME and RME groups were estimated using Hedges’ g correction based on the change between baseline (T0) and post-treatment (T1). A random-effects model with restricted maximum-likelihood estimation was applied to account for between-study heterogeneity.

Forest plots were used to illustrate the individual and pooled effect estimates. Heterogeneity was assessed using Cochran’s Q test and the I^2^ statistic. Publication bias was evaluated using Funnel plots and Egger’s test when applicable. The level of statistical significance was set at 5% (α = 0.05). The software used was R 4.3.1 (R Core Team (2023). R: A language and environment for statistical computing. R Foundation for Statistical Computing, Vienna, Austria. URL http://www.R-project.org/).

## 3. Results

### 3.1. Selection of Sources of Evidence

The electronic database search yielded a total of 511 records, retrieved from PubMed (*n* = 128), Embase (*n* = 151), Scopus (*n* = 100), Web of Science (*n* = 96), and the Cochrane Library (*n* = 36). The manual searching of reference lists did not result in the identification of further studies (*n* = 0).

After the exclusion of duplicate records (*n* = 298), a total of 213 studies were screened by title and abstract. Initial screening led to the exclusion of 196 records because they were not pertinent to the review. Of the 17 full-text articles assessed for eligibility, 13 were excluded primarily due to inconsistencies with the inclusion criteria relating to methodology and appliances.

**Figure 1:**
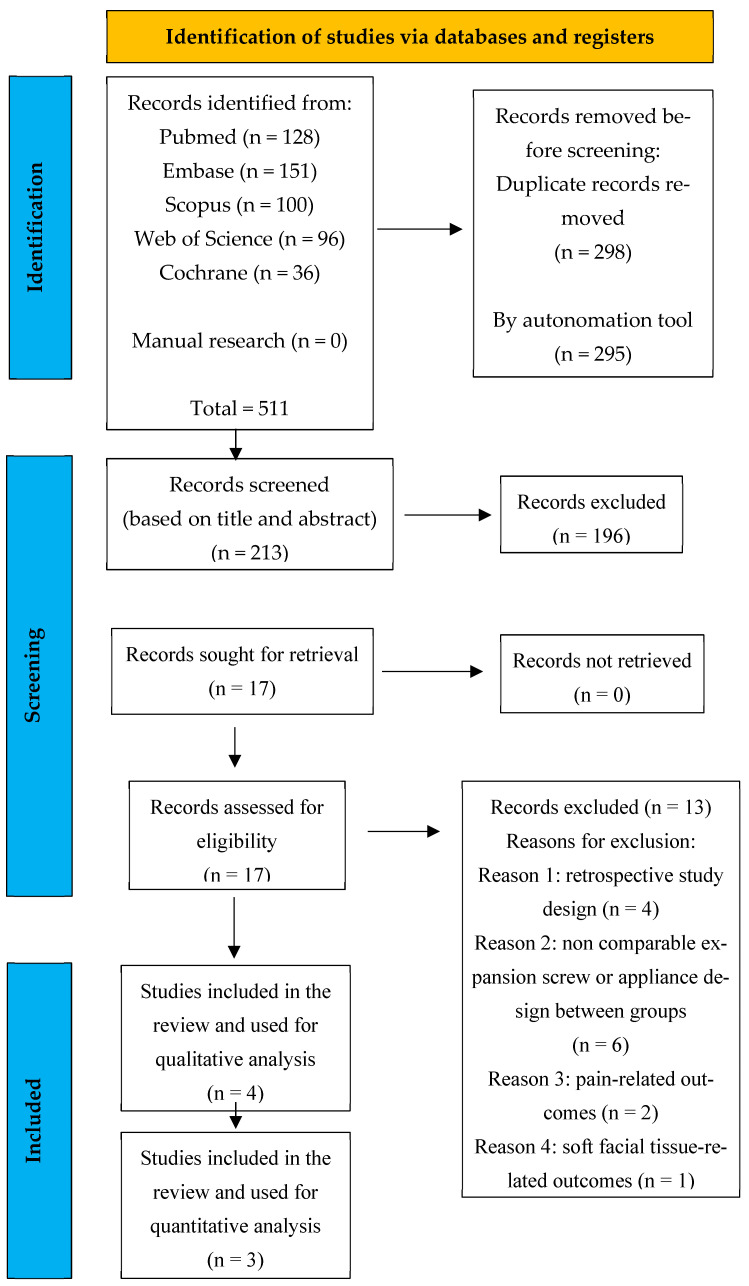
PRISMA flow chart; performed search’s flow diagram.

Details of the study identification and selection process are illustrated in the flow diagram shown in Figure 1.

### 3.2. Characteristics of Sources of Evidence

The four selected studies were all RCTs, published between 2022 and 2025, and conducted in Italy [28,29,30,31]. Most of the included studies were multicenter [28,30,31], and only one was conducted at a single center [29]. The included studies compared various treatment groups [28,29,30], as well as untreated control groups where available [31]. Sample sizes ranged from 32 [29] to 150 patients [30]. Table 2 summarizes the main methodological characteristics of the included studies.

Table 3 shows the clinical characteristics of the enrolled patients.

### 3.3. Intervention Characteristics and Treatment Protocols

The intervention protocols across the four included RCTs were summarized in terms of appliance type, activation regimen, duration of active expansion, and retention strategy. One of the included RCTs [31] adopted a three-arm design including an additional untreated control group; however, the appliance protocol comparison table only considered the SME and RME protocols.

In the SME group, all studies employed an LE appliance with different spring strength patterns. Two trials used an LE of 450 g [29,31], while the remaining studies adopted an LE of 900 g [28,30]. Anchorage was consistently reported as being to the deciduous second primary molars. The LE activation protocol was described as intermittent and clinician-driven, with variability between studies in the initial pre-activation/expansion phase.

In the RME group, the expander was most frequently described as a Hyrax-type rapid maxillary expander. RME activation protocols were usually carried out at home by parents, although some studies involved an initial in-office activation.

The duration of active expansion differed between SME and RME and varied across trials. In the SME group, the reported duration of active treatment ranged from approximately 3 months until dental overcorrection is achieved [28,29,30]; in the RME group, the active expansion period was shorter.

Retention strategies were heterogeneous. Intervention characteristics and treatment protocols are shown in Table 4.

### 3.4. Outcomes

Table 5 and Table 6 summarize the evidence related to the primary and secondary outcomes of this review. The data were divided into two tables to enhance clarity and facilitate consultation.

Table 5 synthesizes the primary outcome of this review. The primary outcomes of the included trials mainly focused on transverse dentoalveolar changes, as assessed using digital dental casts or CBCT-derived landmarks. There was marked heterogeneity in the measurement sites and metrics used, and instances of not reported (NR) data were frequent, particularly regarding midpalatal suture opening and palatal surface changes (the latter of which were only available in one study). Overall, both SME and RME protocols produced transverse increases; however, differences between groups varied according to the anatomical level evaluated.

Secondary outcomes were inconsistently reported. Molar tipping/inclination changes did not show statistically significant differences between protocols in studies providing numerical data, whereas spontaneous upper first molar distorotation was consistently greater with SME in the large RCT by Abate et al. across all subgroups [30]. Table 6 reports the secondary outcomes evaluated.

### 3.5. Risk of Bias Assessment

**Figure 2:**
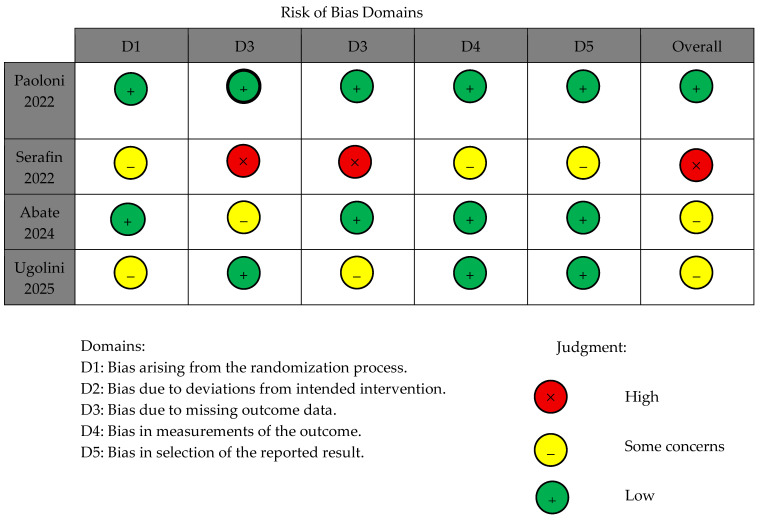
Risk of bias assessment of the included RCTs using the Cochrane RoB 2 tool across five domains [28,29,30,31].

According to the Cochrane RoB 2.0 tool [26], one of the four RCTs was judged to be at low overall risk of bias, two were assessed as presenting some concerns, and one was classified as high risk of bias. Figure 2 illustrates the distribution of risk of bias judgments across the assessed domains.

### 3.6. Quality of Evidence

According to the GRADE framework, the overall certainty of evidence ranged from moderate to very low [27]. Most transverse dental and skeletal width outcomes were rated as low or very low certainty, mainly due to risk of bias, methodological heterogeneity, and imprecision related to small sample sizes or single-study evidence.

Very low certainty was observed for inter-second deciduous molar width, inter-first permanent molar width, basal maxillary width, and upper first molar tipping/inclination, reflecting combined downgrades across multiple GRADE domains. Low certainty was assigned to inter-permanent incisor width, inter-deciduous canine width, and palatal surface changes, primarily because of imprecision and limited replication. Upper first molar distorotation showed the highest certainty (moderate), supported by a large RCT with overall low risk of bias, although downgraded for imprecision due to lack of replication. Table 7 shows the quality of evidence.

### 3.7. Meta-Analysis

Researchers conducted a systematic review of literature, turning out a final selection of four articles. The primary outcome was transverse maxillary change evaluated through skeletal and dental transverse measurements (IDC, ISDM, IFPM, PSOC, PSC, and BMW). However, for each measurement, authors used different dimensions and reference points (e.g., for IDC, Paoloni measured U3-U3 and Abate distance 53–63). Therefore, it is necessary to standardize their results to a uniform scale before combining them. The standardized mean difference (SMD) expresses the size of the intervention effect in each study relative to the variability observed in that study. This approach was considered valid because all measurements reflected the same underlying construct, and the direction of the scale was consistent (larger values meant more expansion). Other considerations to be taken into account are as follows:

• Some authors reported more than one dimension for the same parameter (e.g., Paoloni reported distances U6-U6 and Um-Um regarding IFPM). They will be considered as independent sub-studies.
• Abate reported data differentiating the diagnosis (no cross-bite, unilateral or bilateral). Given that other authors did not differentiate, the averaged mean values will be calculated.
• The outcomes evaluated were inter-canine distance (IDC), inter-second deciduous molar distance (ISDM), inter-first permanent molar distance (IFPM), and basal maxillary width (BMW).

#### 3.7.1. Inter-Canine Distance (IDC)

**Figure 3:**
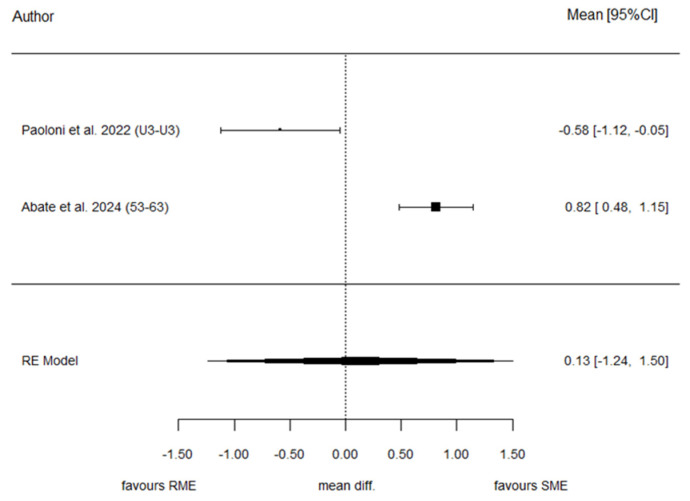
Forest plot of standardized mean differences for IDC between SME and RME groups [28,30].

Two studies provided data suitable for meta-analysis of IDC. The overall effect estimate showed a pooled SMD of 0.13, representing the difference in standardized change between T1 and T0 in the SME and RME groups. No statistically significant difference was observed between the two protocols (*p* = 0.850), indicating comparable effects on inter-canine expansion. We observed that the heterogeneity associated with the model is very high (I^2^ = 94.7%) because both authors reported rather different results. Egger’s test and the Funnel plot were not analyzed because of the low sample size (*n* = 2 studies). Figure 3 shows the forest plot summarizing the effect estimates for IDC.

#### 3.7.2. Inter-Second Deciduous Molar Distance (ISDM)

**Figure 4:**
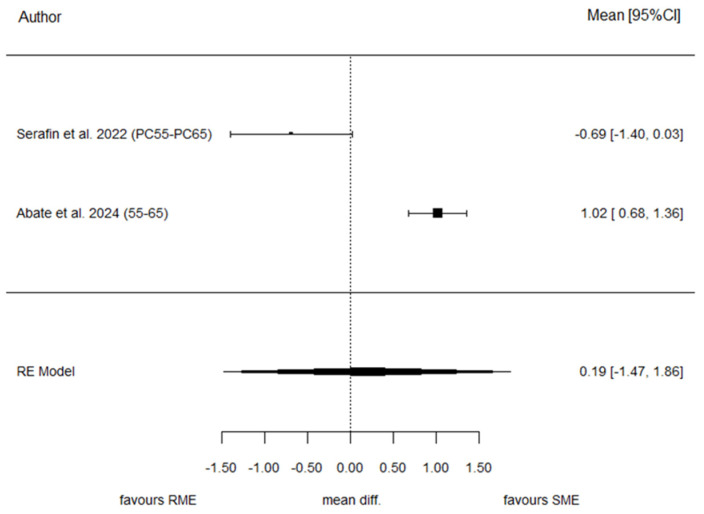
Forest plot of standardized mean differences (SMD) for ISDM comparing SME and RME groups [29,30].

Two studies contributed to the analysis of ISDM. The pooled SMD was 0.19 (95% CI: −1.47 to 1.86), again showing no statistically significant difference between SME and RME (*p* = 0.819). Heterogeneity was also very high for this parameter (I^2^ = 94.4%, *p* < 0.001). Because of the small number of included studies, publication bias assessment was not performed. Figure 4 reports the forest plot for this outcome.

#### 3.7.3. Inter-First Permanent Molar Distance (IFPM)

**Figure 5:**
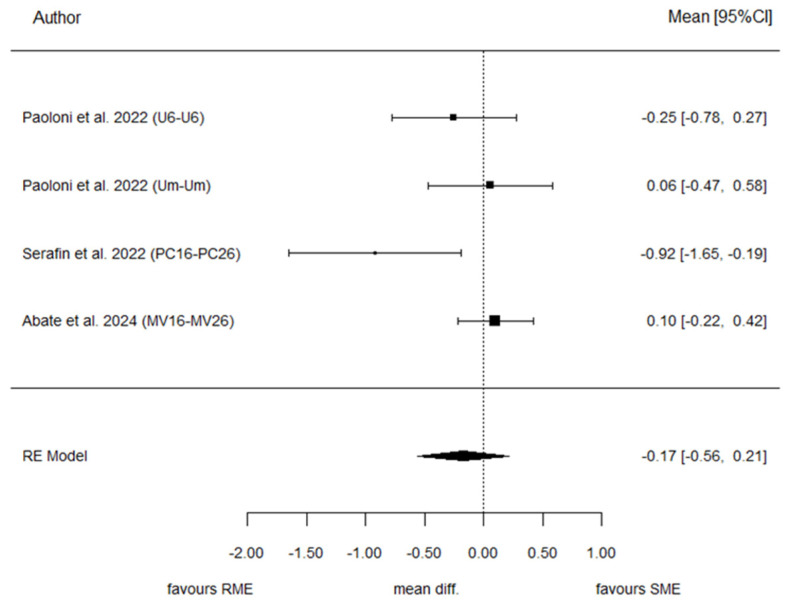
Forest plot of standardized mean differences (SMD) comparing SME and RME for IFPM [28,29,30].

**Figure 6:**
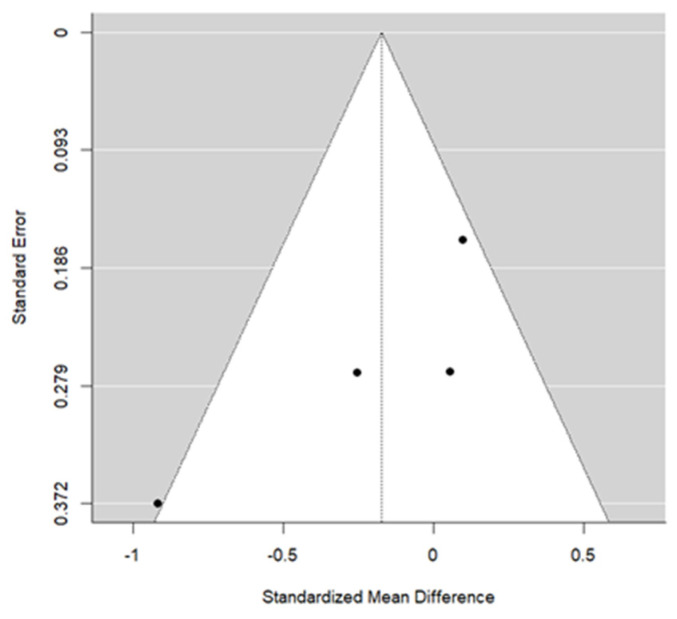
Funnel plot for IFPM used to assess potential publication bias among the included studies.

Three studies reported data for IFPM. The meta-analysis concluded a pooled SMD = −0.17 (this is the difference in standard deviations of the change in T1–T0 between SME and RME groups). This result was not significant (*p* = 0.379), suggesting a similar effect. The heterogeneity associated with the model is moderate (I^2^ = 57.6%). Most articles individually concluded a lack of difference. Cochran’s test of heterogeneity concluded marginal rejection (*p* = 0.074). Egger’s test concluded significance, suggesting a possible bias in publication (*p* = 0.025). Figure 5 and Figure 6 illustrate the forest plot and the funnel plot for IFPM, respectively.

#### 3.7.4. Basal Maxillary Width (BMW)

**Figure 7:**
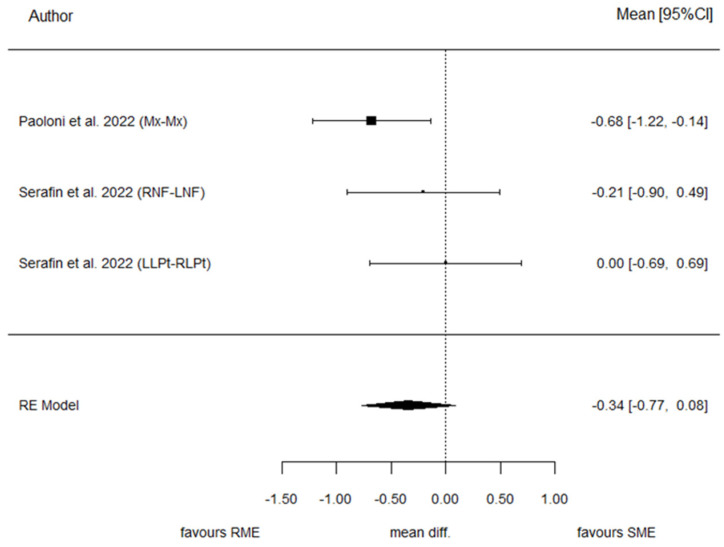
The forest plot for the BMW [28,29].

**Figure 8:**
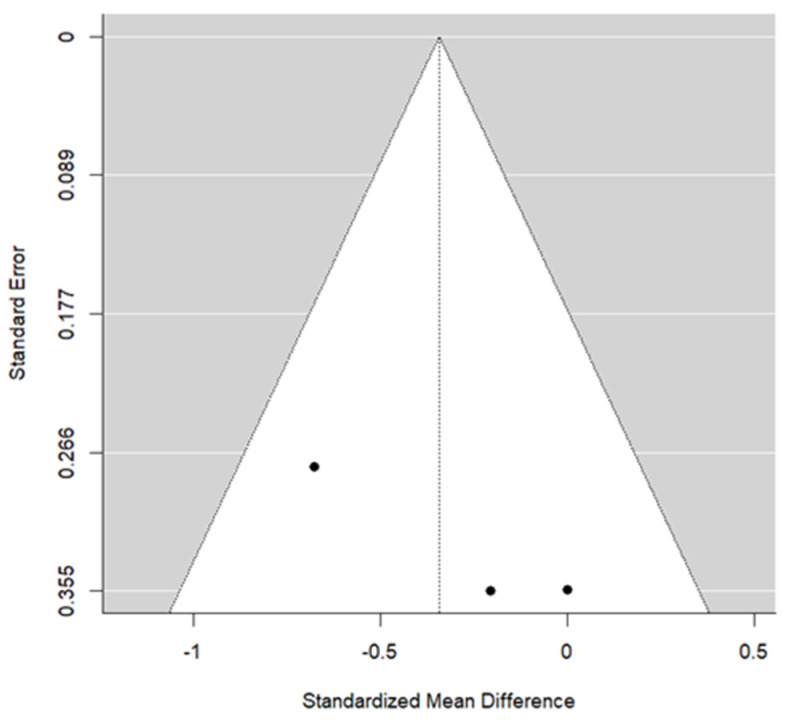
The funnel plot for the BMW.

Two studies provided data for BMW. The pooled effect estimate showed a standardized mean difference (SMD) of −0.34, reflecting the difference in standardized change between T1 and T0 in the SME and RME groups. The result was not statistically significant (*p* = 0.115). This outcome displayed the lowest *p*-value among the evaluated parameters. The heterogeneity associated with the model is low (I^2^ = 26.1%). The overall trend of these articles suggested better results using RME. Cochran’s test of heterogeneity concluded acceptance of homogeneity (*p* = 0.279). Egger’s test did not reach statistical significance (*p* = 0.124). Figure 7 and Figure 8 present the forest plot and the funnel plot for the BMW outcome, respectively.

## 4. Discussion

This systematic review and meta-analysis comparing SME with LE and RME in growing patients indicates that both protocols are effective in correcting transverse maxillary deficiency, producing comparable transverse increases at the molar level, while differences mainly concern the distribution and biomechanical expression of dentoskeletal effects rather than the total amount of expansion achieved.

The quantitative synthesis of the available RCTs further supports these observations. The meta-analysis did not demonstrate statistically significant differences between SME and RME for any of the evaluated transverse parameters, including inter-canine distance (IDC), inter-second deciduous molar distance (ISDM), inter-first permanent molar distance (IFPM), and basal maxillary width (BMW). The pooled standardized mean differences were small and non-significant across outcomes, indicating comparable transverse expansion between the two protocols. However, the meta-analysis also highlighted substantial heterogeneity for some parameters, particularly for IDC and ISDM, reflecting differences in measurement landmarks, imaging modalities, and analytical approaches across studies. In contrast, basal maxillary width showed lower heterogeneity and a tendency toward greater expansion with RME, although this difference did not reach statistical significance.

In the trial by Paoloni et al., expansion effects were assessed using transverse linear measurements on digital dental casts and postero-anterior cephalograms for skeletal width. No significant intergroup differences were found for primary intermolar transverse width, while small but significant advantages for RME were observed at the maxillary inter-canine and skeletal levels [28]. A center–treatment interaction suggested a possible influence of the clinical setting rather than a consistent protocol superiority. Overall, the findings indicate comparable dentoalveolar transverse gains at the first molar level after retention, with only modest anterior dental and skeletal differences between protocols.

Three-dimensional CBCT evidence clarifies the distribution of dental and skeletal effects. Serafin et al. reported significant transverse increases with both protocols when anchored on deciduous teeth, but intergroup comparisons showed greater expansion with RME at the permanent first molars and nasal walls, while other skeletal landmarks, including the pterygoid region, did not differ significantly [29]. A baseline imbalance in molar inclination in the SME group warrants caution in attributing superior tipping control exclusively to the slow protocol. CBCT findings suggest that RME produces a slightly broader anterior skeletal and dental changes, whereas SME achieves clinically meaningful but more localized transverse modifications.

In the two-center trial by Abate et al., three-dimensional digital cast analyses showed spontaneous distorotation of the upper first permanent molars after both protocols, with significantly greater angular changes in the LE groups, irrespective of posterior crossbite status, while permanent intermolar linear width did not differ between treatments [30]. These findings indicate that similar transverse gain at the molar level may arise through different biomechanical mechanisms. In particular, SME appears to be associated with a more rotational component of molar movements. Clinically, spontaneous derotation is advantageous because correction of mesially rotated molars contributes to increased arch perimeter. The greater rotational effect observed with SME may be related to the light and continuous force delivery of the leaf springs and their interaction with anchorage distribution at the deciduous molars. Biomechanically, this phenomenon may be explained by transseptal fiber stretching, which generates a rotational moment as permanent molars follow the displacement of the deciduous teeth [32,33]. Although a similar effect may occur with RME, derotation is typically less pronounced and more closely associated with the opening of the midpalatal suture rather than fiber-mediated adaptation [34].

Ugolini et al. evaluated palatal morphological changes using digital intraoral scans and three-dimensional segmentation analysis [31]. Both the LE and RME groups showed statistically significant increases in total and regional palatal surface area compared with the untreated control group, with the largest increments observed in the median palatal region. However, no statistically significant differences were found between the two active protocols. These findings suggest that when expansion is evaluated as a three-dimensional morphological transformation rather than a single linear dimension, the overall effects of SME and RME appear largely comparable. Although palatal surface area does not represent a direct skeletal measurement, it provides a global morphological perspective that is consistent with linear measurements, indicating similar intermolar expansion between protocols. Some minor reporting inconsistencies were noted in this trial, including the absence of detailed numerical data for certain angular variables and an apparent discrepancy between tabulated and textual descriptions of control-group changes, which were therefore interpreted cautiously [31].

When interpreted in the context of the existing literature, the findings of the included RCTs reveal both concordant trends and some discrepancies. In Paoloni et al., the results partially diverged from the retrospective investigation by Lanteri et al., who did not observe significant intergroup transverse differences [35], and from the findings of Cossellu et al., who reported greater inter-canine increases with the LE and greater intermolar expansion with RME [36]. Conversely, the greater posterior skeletal expansion observed in the RME group by Paoloni was consistent with the conclusions of Rutili et al. [23].

Similarly, Serafin et al. reported that RME tends to be associated with greater overall and skeletal transverse effects than SME, partly attributed to the use of longer expansion screws and a more pronounced orthopedic response when evaluated with three-dimensional imaging [35,37]. However, skeletal-to-dental ratios differed from earlier reports, likely due to differences in landmark selection and measurement protocols [38]. The authors also referred to previous meta-analyses suggesting greater intermolar expansion and skeletal effects with RME, although most of these studies did not use deciduous anchorage, which limits direct comparability [23]. The authors also noted that evidence on molar inclination remains heterogeneous, although deciduous anchorage is generally considered to reduce buccal tipping of permanent molars, and SME may favor dentoalveolar decompensation and sutural remodeling compared with the intermittent force accumulation typical of RME [39]. From a dentoalveolar perspective, Abate et al. confirmed that spontaneous distorotation of the maxillary first molars represents a measurable and clinically relevant phenomenon, in agreement with previous investigations [34], although their findings of greater anterior expansion with the LE contrasted with those reported by Paoloni et al. [28]. Finally, Ugolini et al. emphasized that surface-based analysis may better capture global palatal morphology than isolated linear measurements, which can be influenced by dental tipping [40]. Within this framework, the absence of significant differences between LE and RME surface changes supports the view that SME represents a valid alternative expansion protocol in growing patients [7].

Taken together, current RCT evidence does not demonstrate a clear overall superiority of one protocol in terms of total dentoskeletal transverse expansion. Instead, appliance choice appears to influence the distribution and qualitative pattern of dental and skeletal adaptations rather than the absolute amount of expansion achieved. This interpretation should be considered in light of methodological heterogeneity across studies. Only one trial employed three-dimensional CBCT imaging [29], while others relied on digital casts or surface analyses, limiting direct anatomical comparisons. In addition, standardized direct assessment of midpalatal suture opening was generally lacking. This methodological choice may partly reflect adherence to radiation protection principles, such as the ALARA concept, particularly in pediatric populations [41].

A practical difference between protocols concerns activation modality. The LE is a self-activating appliance, and this contrasts with conventional RME devices, which depend on repeated screw activations performed at home, potentially introducing variability.

In terms of stability, all the included RCTs adopted comparable retention protocols following the active expansion phase [28,29,30,31,32]. Follow-up periods extended up to approximately one year after appliance placement, and both expansion methods showed stable transverse results within this timeframe. However, it remains unclear whether different expansion modalities influence long-term stability once craniofacial growth is completed.

Although patient-reported pain was outside the predefined PICO framework, it remains a clinically relevant aspect of maxillary expansion therapy. RCTs report greater initial discomfort with conventional RME, particularly during the first days of activation [20,42]. This is related to the intermittent orthopedic forces generated during screw activation, which trigger transient inflammatory responses before sutural remodeling occurs [43,44]. Moreover, skeletal expansion represents only part of the total transverse gain, with a relevant contribution from alveolar bending and dental tipping [45]. Although RME is generally well tolerated, analgesic use may occasionally be required during the early activation phase [20,46]. In contrast, SME protocols based on light continuous forces allow more gradual tissue adaptation and may improve treatment acceptance, particularly in pediatric or special-needs patients [21,47].

Three-dimensional facial analyses suggest that RME may produce slightly greater increases in nasal and intercanthal width compared with SME [48]. However, these differences are typically limited to millimetric variations and are unlikely to represent a decisive factor in clinical decision-making.

From a functional perspective, transverse skeletal changes may theoretically influence airway dimensions [49,50]. It is conceivable that the slightly greater skeletal expansion sometimes observed with RME could translate into marginal airflow improvements, whereas the more gradual expansion produced by LE devices might result in less pronounced airway modifications. However, airway function was not directly evaluated in the included RCTs, and therefore any functional inference should be interpreted with caution.

### 4.1. Limitations

Despite providing an updated synthesis of the available randomized evidence, some limitations should be considered when interpreting the results of this review. First, the number of included RCTs was relatively small, and only one trial involved a large sample size [30]. In addition, methodological heterogeneity in outcome definitions, anatomical landmarks, and imaging techniques limited direct comparability across studies. Another limitation concerns the risk of bias: according to the RoB 2 assessment, variability in the methodological quality of the included trials reduces confidence in the available evidence. Another aspect that should be considered is the geographical concentration of the available evidence. All the included RCTs were conducted in Italy between 2022 and 2025, often within closely related academic research networks. While this reflects the strong research interest in leaf-expander protocols within specific orthodontic centers, it may also introduce a degree of regional research clustering. Consequently, the external validity and generalizability of the present findings to other populations and clinical settings should be interpreted with caution. Another important limitation concerns the quantitative synthesis. Although a meta-analysis was performed, the number of studies available for each outcome was small, with some parameters including only two trials. This limited statistical power and reduced the ability to explore publication bias or perform subgroup analyses. Additionally, the included studies used heterogeneous anatomical landmarks and measurement techniques, requiring the use of standardized mean differences to combine results. While this approach allowed quantitative comparison, it also reduced the direct clinical interpretability of the pooled estimates.

### 4.2. Future Perspectives

Future RCTs should aim to strengthen methodological rigor and adopt standardized outcome definitions in order to reduce heterogeneity, selective reporting, and analytical bias. Larger and more geographically diverse populations are also required to improve external validity and generalizability. Future multicenter trials conducted in different countries and healthcare systems would help confirm the reproducibility of these findings and improve the global applicability of the evidence. In addition, future investigations should incorporate objective airway assessments and explore comparisons among different slow expansion appliances or hybrid expansion protocols.

## 5. Conclusions

Within the limitations of the available evidence, both SME performed with spring-based LE and RME performed with conventional Hyrax- or Haas-type appliances appear to be effective in correcting transverse maxillary deficiency in skeletally immature patients. The quantitative meta-analysis confirmed that no statistically significant differences were observed between the two protocols for the main transverse parameters evaluated. The current body of RCTs does not demonstrate a clear overall superiority of one protocol over the other in terms of absolute transverse dento skeletal gain. From a clinical perspective, appliance selection should therefore not be based only on the expectation of greater transverse expansion, but rather on a broader evaluation that includes biomechanical characteristics, patient compliance requirements, comfort, anchorage strategy, and individual treatment objectives. Future high-quality RCTs with standardized 3D measurements, larger and more diverse samples, and longer follow-up (also including objective evaluations of midpalatal suture opening, airway function, and long-term stability) are needed to strengthen the evidence and better clarify the biological and functional effects of different expansion protocols.

## Figures and Tables

**Table 1:** The eligibility criteria used for the review.

Inclusion Criteria	Exclusion Criteria
• RCTs • Skeletally immature patients • Patients with maxillary transverse deficiency and/or posterior crossbite • Comparison between SME using spring-based LE devices and RME using conventional tooth-borne appliances (e.g., Hyrax or Haas-type expanders)	• Non-randomized studies; case series and case reports; reviews, systematic reviews and meta-analyses; animal studies or in vitro studies • Studies not meeting the predefined PICO criteria • Studies including patients with cleft lip and/or palate, with craniofacial syndromes or other craniofacial anomalies, with systemic diseases potentially affecting craniofacial growth

RCTs: randomized clinical trials.

**Table 2:** Main methodological characteristics of the included RCTs.

Authors/ (Year/Country)	Study Design	Study Setting	Group	Sample Size (SME/RME/Control)
Paoloni et al. (2022, Italy) [28]	RCT	Multicentre (2 centres)	SME v.s RME	56 (28/28/NA)
Serafin et al. (2022, Italy) [29]	RCT	Single centre	SME vs. RME	32 (16/16/NA)
Abate et al. (2025, Italy) [30]	RCT	Multicentre (2 centres)	SME vs. RME	150 (75/75/NA)
Ugolini et al. (2025, Italy) [31]	RCT	Multicentre (2 centres)	SME vs. RME vs. control group	63 (24/22/17)

SME: slow maxillary expansion; RME: rapid maxillary expansion; RCT: randomized clinical trial; NA: not applicable.

**Table 3:** Clinical characteristics and diagnostic criteria of the enrolled patients in the included studies.

Studies	Gender (SME/RME/Control)	Age (SME/RME/Control)	Cervical Vertebral Maturation	Type of Malocclusion	Stage Dentition	Diagnostic Criteria
Paoloni et al. [28]	17 F; 11 M/ 12 F; 16 M/ NA	8.0 ± 1.3 y/ 8.4 ± 1.0 y/ NA	CS1-CS2	NR	Early or intermediate mixed dentition stage, fully erupted upper and lower first permanent molars and presence of the second upper deciduous molars available as an anchoring teeth	Posterior transverse interarch discrepancy ≥ 3 mm (dental casts)
Serafin et al. [29]	5 F; 11 M/ 12 F; 6 M/ NA	8.96 ± 0.92 y/ 8.50 ± 1.39 y/ NA	NR	Class I–II with or without posterior crossbite	Early mixed dentition stage, erupted upper and lower first permanent molars and non-mobile maxillary deciduous second molars	Maxillary transverse discrepancy (CBCT basal bone measurements at first molars)
Abate et al. [30]	43 F; 32 M/ 40 F; 35 M/ NA	7.84 ± 0.5 y/ 7.68 ± 0.92 y/ NA	CS1-CS2	Unilateral posterior cross bite (*n* = 57); bilateral posterior crossbite (*n* = 32); without crossbite (*n* = 61)	Mixed dentition with fully erupted permanent upper first molars and maxillary deciduous second molars available for anchorage	Posterior transverse discrepancy ≥ 3 mm (digital dental models)
Ugolini et al. [31]	11 F; 13 M/ 12 F; 10 M/ 10 F; 7 M	8.5 ± 1.5 y/ 7.9 ± 1.6 y/ 8.1 ± 1.2 y	CS less than 3	Bilateral posterior crossbite (*n* = 8)	Mixed dentition with fully erupted upper and lower first molars and upper second molar available for anchorage	Intermolar width < 30 mm

SME: slow maxillary expansion; RME: rapid maxillary expansion; F: females; M: males; NA: not applicable; CS: cervical stage; NR: not reported; CBCT: cone beam computed tomography.

**Table 4:** Intervention characteristics and treatment protocols of the included RCTs.

Study	SME Appliance	SME Activation Protocol	RME Appliance	RME Activation Protocol *	Active Expansion Duration	Retention/Appliance In Situ after Activation
Paoloni et al. [28]	LE 900 g (anchored on second primary molars)	Initial expansion 4.5 mm in 2–3 months; clinician activation monthly (15 quarter-turns/month)	Conventional rapid maxillary expander (anchored on second primary molars)	1/4 turn per day (0.2 mm per activation) activated by parents	4.5 ± 1.1 months (SME); 1.0 ± 0.4 months (RME)	Both groups removed 1 year after application
Serafin et al. [29]	LE 450 g (anchored to deciduous molars)	Pre-activated 3 mm in laboratory; clinician reactivation for 3 times (1 mm per month)	Hyrax expander (anchored on second primary molars)	Initial 2 turns chairside (=0.50 mm); then 1 turn/day (0.25 mm) by parents	3 months until dental overcorrection is achieved (SME); until dental overcorrection is achieved (RME)	Passive in situ for 6 months after active expansion (SME); passive in situ for 7 months after active expansion (RME)
Abate et al. [30]	LE 900 g (anchored to deciduous molars)	Initial pre-activation 3/4.5 mm in 2–3 months; clinical activation monthly (10/15 quarter-turns/month) until desired expansion reached	Hyrax expander (anchored on second primary molars)	1/4 turn twice a day (0.45 mm activation per day) until dental overcorrection	Until the desired expansion was reached (SME); 10 ± 2 days (RME)	Both groups maintained passively for a minimum of 6 months
Ugolini et al. [31]	LE 450 g (anchored to deciduous molars)	0.1 mm/turn (10 turns = 1 mm); max 30–45 activations depending on screw size based on the patient’s transverse discrepancy	Hyrax expander (anchored on second primary molars)	1/4 turn twice a day (0.45 mm activation per day) until dental overcorrection	NR (SME); 10 ± 2 days (RME)	NR; passive retention ≥ 6 months (RME)

SME: slow maxillary expansion; RME: rapid maxillary expansion; LE: leaf expander; NR: not reported. * Activation values are reported as described in the original studies. Linear equivalents are provided where explicitly stated.

**Table 5:** Primary outcomes.

Study	Inter Permanent Incisive Width Change from T0 to T1 (mm) SME/RME (*p* Value)	Inter Deciduous Canine Width Change from T0 to T1 (mm) SME/RME (*p* Value)	Inter Secondary Deciduous Molar Width Change from T0 to T1 (mm) SME/RME (*p* Value)	Inter First Permanent Molar Width Change from T0 to T1 (mm) SME/RME (*p* Value)	Palatal Suture Opening Change from T0 to T1 (mm) SME/RME (*p* Value)	Palatal Surface Changes Change from T0 to T1 (mm^2^) SME/RME (*p* Value)	Basal Maxillary Width Change from T0 to T1 (mm) SME/RME (*p* Value)
Paoloni et al. [28]	NR	U3-U3: +2.2 ± 1.4/+3.0 ± 1.3 (*p* = 0.005)	NR	U6-U6: +4.1 ± 1.7 mm/ +4.5 ± 1.4 mm (*p* = 0.365); Um-Um: +3.3 ± 1.9 mm/+3.2 ± 1.6 mm (*p* = 0.889)	NR	NR	Mx-Mx: +2.3 ± 1.6/+3.7 ± 2.4 (*p* = 0.013)
Serafin et al. [29]	PC11_PC21: 0.0 ± 1.4/0.8 ± 0.7 (*p* = 0.058)	NR	PC55_PC65: 4.5 ± 2.4/6.3 ± 2.7 (*p* = 0.790)	PC16_PC26: 2.2 ± 1.7/3.9 ± 1.9 (*p* = 0.025)	NR	NR	RNF_LNF: 0.9 ± 2.3/1.7 ± 4.8 (*p* = 0.184); LLPt_RLPt: 1.3 ± 1.3/1.3 ± 1.8 (*p* = 0.849)
Abate et al. [30]	NR	53–63: No-crossbite: 5.36 ± 2.02/4.02 ± 2.01 (*p* = 0.01) Unilateral-crossbite: 5.20 ± 1.61/3.42 ± 1.43 (*p* < 0.01) Bilateral-crossbite: 5.68 ± 1.60/4.71 ± 1.33 (*p* = 0.05)	55–65: No-crossbite: 5.52 ± 1.39/4.47 ± 1.57 (*p* = 0.008) Unilateral-crossbite: 5.41 ± 1.99/4.22 ± 1.54 (*p* = 0.014) Bilateral-crossbite: 7.16 ± 1.17/5.23 ± 1.37 (*p* < 0.01)	MV16-MV26: No-crossbite: 3.42 ± 1.16/4.13 ± 1.77 (*p* = 0.07) Unilateral-crossbite: 4.60 ± 1.43/4.26 ± 1.86 (*p* = 0.43) Bilateral-crossbite: 5.44 ± 1.68/4.64 ± 1.33 (*p* = 0.12)	NR	NR	NR
Ugolini et al. [31]	NR	NR	NR	NR	NR	Total Palatal Surface: 155.4 ± 49.9/187.7 ± 58.0 (*p* = 0.0612)	NR

SME: slow maxillary expansion; RME: rapid maxillary expansion; NR: note reported; U3-U3: maxillary inter-canine width (distance between the tips of the cusps of right and left maxillary deciduous canines); U6-U6: maxillary intermolar width (distance between the central fossae of right and left maxillary first permanent molars); Um-Um: maxillary intermolar width (distance between the most prominent lateral point on the buccal surface of the maxillary first permanent molars); Mx-Mx: maxillary skeletal width (distance between the most prominent lateral points on the buccal surfaces of the maxillary first permanent molars); PC11_PC21: central incisors width (distance between the pulp chamber centers of the upper right and left permanent central incisors); PC55_PC65: deciduous molars width (distance between the pulp chamber centers of the upper right and left second deciduous molars); PC16_PC26: permanent molars width (distance between the pulp chamber centers of the upper right and left first permanent molars); RNF_LNF: nasal floor width (transverse distance between the right and left nasal floor cortical junctions, representing anterior skeletal maxillary width, junction between palatal cortical bone and nasal cavity cortical bone); LLPt_RLPt: lateral pterygoid width (lateral pterygoid transverse distance between the left and right lateral pterygoid plates, representing posterior skeletal maxillary width); 53–63: upper inter-canine width (measured at the cusp tips of the right and left deciduous canines on digital dental casts); 55–65: upper inter-deciduous second deciduous molar width (measured at the cusp tips of the right and left deciduous second molars on digital dental casts); MV16-MV26: upper intermolar width (measured at the mesiobuccal cusp tips of the right and left first permanent molars on digital dental casts).

**Table 6:** Secondary outcomes.

Study	Upper First Molar Distorotation Change from T0 to T1 (°) SME/RME (*p* Value)	Molar Tipping Change from T0 to T1 (°) SME/RME (*p* Value)
Paoloni et al. [28]	NR	U6 BLI: −7.1 ± 5.8°/−3.5 ± 3.8° (*p* = 0.077)
Serafin et al. [29]	NR	FURCA16_PC16_PC26 (◦): −2.9 ± 11.9/−0.6 ± 18.7 (*p* = 0.323); FURCA26_PC26_PC16 (◦): 1.3 ± 8.4/0.8 ± 25.8 (*p* = 0.879)
Abate et al. [30]	No-crossbite: D16: −6.24 ± 3.52/−4.17 ± 2.84 (*p* = 0.014); D26: −5.91 ± 3.26/−4.08 ± 2.10 (*p* = 0.012). Unilateral-crossbite: D16: −6.91 ± 3.89/−3.63 ± 2.54 (*p* < 0.01); D26: −6.68 ± 5.11/−4.35 ± 3.01 (*p* = 0.044). Bilateral-crossbite: D16: −6.97 ± 3.75/4.50 ± 2.45 (*p* = 0.027); D26: −5.10 ± 2.94/2.71 ± 1.80 (*p* = 0.007).	NR
Ugolini et al. [31]	NR	Greater permanent molar inclination qualitatively reported in the RME group; no numerical data provided

SME: slow maxillary expansion; RME: rapid maxillary expansion; NR: not reported; U6 BLI: mean value for BLI between right and left U6; FURCA16_PC16_PC26 (◦): right upper molar tip; FURCA26_PC26_PC16 (◦): left upper molar tip; D16: angle formed by the intersection of the mid palatal plane with the planes P16 (passing through disto vestibular cusps of upper first permanent right molar and mesio palatal cusps of upper first permanent right molar); D26: angle formed by the intersection of the mid palatal plane with the planes P26 (passing through disto vestibular cusps of upper first permanent left molar and mesio palatal cusps of upper left permanent right molar).

**Table 7:** Summary of the quality of evidence (GRADE) for the main outcomes evaluated.

Outcome	N° Studies	Risk of Bias	Inconsistency	Indirectness	Imprecision	Publication Bias	Certainty
Inter permanent incisor width	1	Serious	Not applicable	Not serious	Serious	Not assessed	LOW
Inter deciduous canine width	2	Not serious	Serious	Not serious	Serious	Not assessed	LOW
Inter second deciduous molar width	2	Serious	Serious	Not serious	Serious	Not assessed	VERY LOW
Inter first permanent molar width	3	Serious	Serious	Serious	Serious	Not assessed	VERY LOW
Basal maxillary width	2	Serious	Serious	Serious	Serious	Not assessed	VERY LOW
Palatal surface changes	1	Serious	Not applicable	Not serious	Serious	Not assessed	LOW
Upper first molar tipping/inclination	2	Serious	Serious	Serious	Serious	Not assessed	VERY LOW
Upper first molar distorotation	1	Not serious	Not applicable	Not serious	Serious	Not assessed	MODERATE

## Data Availability

The data presented in this study are available in the article.

## Supplementary Materials

The following supporting information can be downloaded at https://www.mdpi.com/article/10.3390/children13030396/s1: Table S1: Search strategy for the five databases.

## References

[1] Thilander B., Wahlund S., Lennartsson B. (1984). The effect of early interceptive treatment in children with posterior cross-bite. Eur. J. Orthod..

[2] De Ridder L., Aleksieva A., Willems G., Declerck D., Cadenas de Llano-Pérula M. (2022). Prevalence of orthodontic malocclusions in healthy children and adolescents: A systematic review. Int. J. Environ. Res. Public Health.

[3] Lione R., Franchi L., Cozza P. (2013). Does rapid maxillary expansion induce adverse effects in growing subjects?. Angle Orthod..

[4] Ronsivalle V., Isola G., Lo Re G., Boato M., Leonardi R., Lo Giudice A. (2023). Analysis of maxillary asymmetry before and after treatment of functional posterior cross-bite: A retrospective study using 3D imaging system and deviation analysis. Prog. Orthod..

[5] Baccetti T., Franchi L., McNamara J.A., Tollaro I. (1997). Early dentofacial features of Class II malocclusion: A longitudinal study from the deciduous through the mixed dentition. Am. J. Orthod. Dentofac. Orthop..

[6] McNamara J.A. (2000). Maxillary transverse deficiency. Am. J. Orthod. Dentofac. Orthop..

[7] Bucci R., D’Antò V., Rongo R., Valletta R., Martina R., Michelotti A. (2016). Dental and skeletal effects of palatal expansion techniques: A systematic review of the current evidence from systematic reviews and meta-analyses. J. Oral Rehabil..

[8] Aprile M., Verdecchia A., Dettori C., Spinas E. (2025). Malocclusion and its relationship with sound speech disorders in deciduous and mixed dentition: A scoping review. Dent. J..

[9] Ureni R., Verdecchia A., Suárez-Fernández C., Mereu M., Schirru R., Spinas E. (2024). Effectiveness of elastodontic devices for correcting sagittal malocclusions in mixed dentition patients: A scoping review. Dent. J..

[10] Haas A.J. (1961). Rapid expansion of the maxillary dental arch and nasal cavity by opening the midpalatal suture. Angle Orthod..

[11] Wertz R.A. (1970). Skeletal and dental changes accompanying rapid midpalatal suture opening. Am. J. Orthod..

[12] Kanomi R., Deguchi T., Kakuno E., Takano-Yamamoto T., Roberts W.E. (2013). CBCT of skeletal changes following rapid maxillary expansion to increase arch-length with a development-dependent bonded or banded appliance. Angle Orthod..

[13] Zimring J.F., Isaacson R.J. (1965). Forces produced by rapid maxillary expansion. III. Forces present during retention. Angle Orthod..

[14] Cameron C.G., Franchi L., Baccetti T., McNamara J.A. (2002). Long-term effects of rapid maxillary expansion: A posteroanterior cephalometric evaluation. Am. J. Orthod. Dentofac. Orthop..

[15] Colino-Gallardo P., Del Fresno-Aguilar I., Castillo-Montaño L., Colino-Paniagua C., Baptista-Sánchez H., Criado-Pérez L., Alvarado-Lorenzo A. (2023). Skeletal and dentoalveolar changes in growing patients treated with rapid maxillary expansion measured in 3D cone-beam computed tomography. Biomedicines.

[16] Liu S., Xu T., Zou W. (2015). Effects of rapid maxillary expansion on the midpalatal suture: A systematic review. Eur. J. Orthod..

[17] Proffit W.R., Fields H.W., Sarver D.M. (2019). Contemporary Orthodontics.

[18] Gracco A., Malaguti A., Lombardo L., Mazzoli A., Raffaeli R. (2010). Palatal volume following rapid maxillary expansion in mixed dentition. Angle Orthod..

[19] Lo Giudice A., Barbato E., Cosentino L., Ferraro C.M., Leonardi R. (2018). Alveolar bone changes after rapid maxillary expansion with tooth-born appliances: A systematic review. Eur. J. Orthod..

[20] Ugolini A., Cossellu G., Farronato M., Silvestrini-Biavati A., Lanteri V. (2020). A multicenter, prospective, randomized trial of pain and discomfort during maxillary expansion: Leaf expander versus hyrax expander. Int. J. Paediatr. Dent..

[21] Lanteri C., Beretta M., Lanteri V., Gianolio A., Cherchi C., Franchi L. (2016). The Leaf Expander for non-compliance treatment in the mixed dentition. J. Clin. Orthod..

[22] Silvestrini-Biavati F., Imenpour S., Poli F., Kola E., Abate A., Lanteri V., Ugolini A. (2024). Three-dimensional analysis of maxillary expansion during rapid and slow protocols. Appl. Sci..

[23] Rutili V., Mrakic G., Nieri M., Franceschi D., Pierleoni F., Giuntini V., Franchi L. (2021). Dento-skeletal effects produced by rapid versus slow maxillary expansion using fixed jackscrew expanders: A systematic review and meta-analysis. Eur. J. Orthod..

[24] Page M.J., McKenzie J.E., Bossuyt P.M., Boutron I., Hoffmann T.C., Mulrow C.D., Shamseer L., Tetzlaff J.M., Akl E., Brennan S.E. (2021). The PRISMA 2020 statement: An updated guideline for reporting systematic reviews. BMJ.

[25] Cohen J. (1960). A coefficient of agreement for nominal scales. Educ. Psychol. Meas..

[26] Sterne J.A.C., Savović J., Page M.J., Elbers R.G., Blencowe N.S., Boutron I., Cates C.J., Cheng H.Y., Corbett M.S., Eldridge S.M. (2019). RoB 2: A revised tool for assessing risk of bias in randomised trials. BMJ.

[27] Guyatt G.H., Oxman A.D., Vist G.E., Kunz R., Falck-Ytter Y., Alonso-Coello P., Schünemann H.J. (2008). GRADE: An emerging consensus on rating quality of evidence and strength of recommendations. BMJ.

[28] Paoloni V., Giuntini V., Lione R., Nieri M., Barone V., Marino Merlo M., Mazza F., Passaleva S., Cozza P., Franchi L. (2022). Comparison of the dento-skeletal effects produced by Leaf expander versus rapid maxillary expander in prepubertal patients: A two-center randomized controlled trial. Eur. J. Orthod..

[29] Serafin M., Fastuca R., Caprioglio A. (2022). CBCT analysis of dento-skeletal changes after rapid versus slow maxillary expansion on deciduous teeth: A randomized clinical trial. J. Clin. Med..

[30] Abate A., Ugolini A., Bruni A., Quinzi V., Lanteri V. (2025). Three-dimensional assessment on digital cast of spontaneous upper first molar distorotation after Ni-Ti leaf springs expander and rapid maxillary expander: A two-centre randomized controlled trial. Orthod. Craniofac. Res..

[31] Ugolini A., Bruni A., Abate A., Pistoni F., Donelli M., Quinzi V., Silvestrini-Biavati F., Lanteri V. (2025). Effects on palatal surface area in mixed dentition patients treated with leaf expander and rapid palatal expander, compared with untreated subjects: A randomized clinical trial. Eur. J. Paediatr. Dent..

[32] Rosa M., Lucchi P., Manti G., Caprioglio A. (2016). Rapid palatal expansion in the absence of posterior cross-bite to intercept maxillary incisor crowding in the mixed dentition: A CBCT evaluation of spontaneous changes of untouched permanent molars. Eur. J. Paediatr. Dent..

[33] Bruni A., Abate A., Maspero C., Castroflorio T. (2024). Comparison of mechanical behavior of clear aligner and rapid palatal expander on transverse plane: An in vitro study. Bioengineering.

[34] Cerruto C., Ugolini A., Vece L., Doldo T., Caprioglio A., Silvestrini-Biavati A. (2017). Cephalometric and dental arch changes to Haas-type rapid maxillary expander anchored to deciduous vs permanent molars: A multicenter randomized controlled trial. J. Orofac. Orthop..

[35] Lanteri V., Cossellu G., Gianolio A., Beretta M., Lanteri C., Cherchi C., Farronato G. (2018). Comparison between RME, SME and Leaf Expander in growing patients: A retrospective postero-anterior cephalometric study. Eur. J. Paediatr. Dent..

[36] Cossellu G., Ugolini A., Beretta M., Farronato M., Gianolio A., Maspero C., Lanteri V. (2020). Three-dimensional evaluation of slow maxillary expansion with leaf expander vs. rapid maxillary expansion in a sample of growing patients: Direct effects on maxillary arch and spontaneous mandibular response. Appl. Sci..

[37] Luiz Ulema Ribeiro G., Jacob H.B., Brunetto M., da Silva Pereira J., Motohiro Tanaka O., Buschang P.H. (2020). A preliminary 3-D comparison of rapid and slow maxillary expansion in children: A randomized clinical trial. Int. J. Paediatr. Dent..

[38] Lo Giudice A., Fastuca R., Portelli M., Militi A., Bellocchio M., Spinuzza P., Briguglio F., Caprioglio A., Nucera R. (2017). Effects of rapid vs. slow maxillary expansion on nasal cavity dimensions in growing subjects: A methodological and reproducibility study. Eur. J. Paediatr. Dent..

[39] Alves A.C.M., Maranhão O.B.V., Janson G., Garib D.G. (2017). Mandibular dental arch short- and long-term spontaneous dentoalveolar changes after slow or rapid maxillary expansion: A systematic review. Dent. Press J. Orthod..

[40] Primožic J., Baccetti T., Franchi L., Richmond S., Farčnik F., Ovsenik M. (2013). Three-dimensional assessment of palatal change in a controlled study of unilateral posterior crossbite correction in the primary dentition. Eur. J. Orthod..

[41] Firetto M.C., Abbinante A., Barbato E., Bellomi M., Biondetti P., Borghesi A., Bossu’ M., Cascone P., Corbella D., Di Candido V. (2019). National guidelines for dental diagnostic imaging in the developmental age. Radiol. Med..

[42] Nieri M., Paoloni V., Lione R., Barone V., Marino Merlo M., Giuntini V., Cozza P., Franchi L. (2021). Comparison between two screws for maxillary expansion: A multicenter randomized controlled trial on patient’s reported outcome measures. Eur. J. Orthod..

[43] Melsen B. (1975). Palatal growth studied on human autopsy material. A histologic microradiographic study. Am. J. Orthod..

[44] Feldmann I., Bazargani F. (2017). Pain and discomfort during the first week of rapid maxillary expansion using two different RME appliances: A randomized controlled trial. Angle Orthod..

[45] Bazargani F., Feldmann I., Bondemark L. (2013). Three-dimensional analysis of effects of rapid maxillary expansion on facial sutures and bones. Angle Orthod..

[46] Baldini A., Nota A., Santariello C., Assi V., Ballanti F., Cozza P. (2015). Influence of activation protocol on perceived pain during rapid maxillary expansion. Angle Orthod..

[47] Rutili V., Nieri M., Franceschi D., Pierleoni F., Giuntini V., Franchi L. (2022). Comparison of rapid versus slow maxillary expansion on patient-reported outcome measures in growing patients: A systematic review and meta-analysis. Prog. Orthod..

[48] Marino Merlo M., Quiroga Souki B., Nieri M., Bonanno A., Giuntini V., McNamara J.A., Franchi L. (2024). Comparison of the effects on facial soft tissues produced by rapid and slow maxillary expansion using stereophotogrammetry: A randomized clinical trial. Prog. Orthod..

[49] Benetti M., Montresor L., Cantarella D., Zerman N., Spinas E. (2024). Does miniscrew-assisted rapid palatal expansion influence upper airway in adult patients? A scoping review. Dent. J..

[50] Abdalla Y., Sonnesen L. (2024). Association between orthodontic treatment and upper airway changes in children assessed with cone-beam computed tomography: A systematic review. J. Oral Rehabil..

