# Supplementary material for: Transverse Maxillary Correction: Leaf Expander vs. Rapid Maxillary Expansion Appliances—A Systematic Review and Meta-Analysis
Source: Children (Basel). 2026 Mar 12;13(3):396. doi: 10.3390/children13030396 (PMC13025118; doi:10.3390/children13030396)
Supplement: Supplementary file 1 [file children-13-00396-s001.zip › children-4186360-supplementary.pdf]

Table S1. Search strategy for the five databases.

Database	Search Strategy
PubMed	((("plant leaves"[MeSH Terms] OR ("plant"[All Fields] AND "leaves"[All Fields]) OR "plant leaves"[All Fields] OR "leaf"[All Fields]) AND ("expander"[All Fields] OR "expanders"[All Fields])) OR (("plant leaves"[MeSH Terms] OR ("plant"[All Fields] AND "leaves"[All Fields]) OR "plant leaves"[All Fields] OR "leaf"[All Fields]) AND ("ego"[MeSH Terms] OR "ego"[All Fields] OR "self"[All Fields]) AND ("expander"[All Fields] OR "expanders"[All Fields])) OR ("slow"[All Fields] AND ("palatal expansion technique"[MeSH Terms] OR ("palatal"[All Fields] AND "expansion"[All Fields] AND "technique"[All Fields]) OR "palatal expansion technique"[All Fields] OR ("maxillary"[All Fields] AND "expansion"[All Fields]) OR "maxillary expansion"[All Fields]))) AND ("RME"[All Fields] OR ("hyraxes"[MeSH Terms] OR "hyraxes"[All Fields] OR "hyrax"[All Fields]) OR "Haas"[All Fields] OR (("rapid"[All Fields] OR "rapidities"[All Fields] OR "rapidity"[All Fields] OR "rapidness"[All Fields]) AND ("palatal expansion technique"[MeSH Terms] OR ("palatal"[All Fields] AND "expansion"[All Fields] AND "technique"[All Fields]) OR "palatal expansion technique"[All Fields] OR ("maxillary"[All Fields] AND "expansion"[All Fields]) OR "maxillary expansion"[All Fields])) OR ((("maxilla"[MeSH Terms] OR "maxilla"[All Fields] OR "maxillary"[All Fields] OR "maxillaries"[All Fields] OR "maxillaris"[All Fields]) AND ("transversal"[All Fields] OR "transversally"[All Fields] OR "transversals"[All Fields] OR "transverse"[All Fields] OR "transversed"[All Fields] OR "transversely"[All Fields] OR "transverses"[All Fields] OR "transversing"[All Fields]) AND ("deficiences"[All Fields] OR "deficiencies"[All Fields] OR "deficiency"[MeSH Subheading] OR "deficiency"[All Fields] OR "deficient"[All Fields] OR "deficients"[All Fields])) OR (("maxilla"[MeSH Terms] OR "maxilla"[All Fields] OR "maxillary"[All Fields] OR "maxillaries"[All Fields] OR "maxillaris"[All Fields]) AND ("transversal"[All Fields] OR "transversally"[All Fields] OR "transversals"[All Fields] OR "transverse"[All Fields] OR "transversed"[All Fields] OR "transversely"[All Fields] OR "transverses"[All Fields] OR "transversing"[All Fields]) AND ("abnormalities"[MeSH Subheading] OR "abnormalities"[All Fields] OR "hypoplasia"[All Fields] OR "hypoplasias"[All Fields])) OR (("maxilla"[MeSH Terms] OR "maxilla"[All Fields] OR "maxillary"[All Fields] OR "maxillaries"[All Fields] OR "maxillaris"[All Fields]) AND ("transversal"[All Fields] OR "transversally"[All Fields] OR "transversals"[All Fields] OR "transverse"[All Fields] OR "transversed"[All Fields] OR "transversely"[All Fields] OR "transverses"[All Fields] OR "transversing"[All Fields]) AND ("discrepancies"[All Fields] OR "discrepancy"[All Fields] OR "discrepant"[All Fields] OR "discrepants"[All Fields] OR "discrepency"[All Fields])) OR (("posterior"[All Fields] OR "posteriors"[All Fields]) AND ("malocclusion"[MeSH Terms] OR "malocclusion"[All Fields] OR "crossbite"[All Fields] OR "crossbites"[All Fields]))))

Embase	(('plant leaf'/exp OR ('plant' AND 'leaves') OR 'plant leaves' OR 'leaf') AND ('expander' OR 'expanders') OR (('plant leaf'/exp OR ('plant' AND 'leaves') OR 'plant leaves' OR 'leaf') AND ('ego'/exp OR 'ego' OR 'self') AND ('expander' OR 'expanders')) OR ('slow' AND ('palatal expansion'/exp OR ('palatal' AND 'expansion' AND 'technique') OR 'palatal expansion technique' OR ('maxillary' AND 'expansion') OR 'maxillary expansion'))) AND ('rme' OR 'hyrax'/exp OR 'hyraxes' OR 'hyrax' OR 'haas' OR (('rapid' OR 'rapidities' OR 'rapidity' OR 'rapidness') AND ('palatal expansion'/exp OR ('palatal' AND 'expansion' AND 'technique') OR 'palatal expansion technique' OR ('maxillary' AND 'expansion') OR 'maxillary expansion')) OR (('maxilla'/exp OR 'maxilla' OR 'maxillary' OR 'maxillaries' OR 'maxillaris') AND ('transversal' OR 'transversally' OR 'transversals' OR 'transverse' OR 'transversed' OR 'transversely' OR 'transverses' OR 'transversing') AND ('deficiences' OR 'deficiencies' OR 'deficiency' OR 'deficient' OR 'deficients')) OR (('maxilla'/exp OR 'maxilla' OR 'maxillary' OR 'maxillaries' OR 'maxillaris') AND ('transversal' OR 'transversally' OR 'transversals' OR 'transverse' OR 'transversed' OR 'transversely' OR 'transverses' OR 'transversing') AND ('abnormalities' OR 'hypoplasia' OR 'hypoplasias')) OR (('maxilla'/exp OR 'maxilla' OR 'maxillary' OR 'maxillaries' OR 'maxillaris') AND ('transversal' OR 'transversally' OR 'transversals' OR 'transverse' OR 'transversed' OR 'transversely' OR 'transverses' OR 'transversing') AND ('discrepancies' OR 'discrepancy' OR 'discrepant' OR 'discrepants' OR 'discrepency')) OR (('posterior' OR 'posteriors') AND ('malocclusion'/exp OR 'malocclusion' OR 'crossbite' OR 'crossbites')))
Scopus	TITLE-ABS-KEY (("leaf expander" OR "leaf self expander" OR "slow maxillary expansion") AND ((RME OR Hyrax OR Haas OR "rapid maxillary expansion") OR ("maxillary transverse deficiency" OR "maxillary transverse hypoplasia" OR "maxillary transverse discrepancy" OR "posterior crossbite")))
Web of Science	TS = (("leaf expander" OR "leaf self expander" OR "slow maxillary expansion") AND ((RME OR Hyrax OR Haas OR "rapid maxillary expansion") OR ("maxillary transverse deficiency" OR "maxillary transverse hypoplasia" OR "maxillary transverse discrepancy" OR "posterior crossbite")))
Cochrane Library	(("leaf expander" OR "leaf self expander" OR "slow maxillary expansion"):ti,ab) AND ((RME OR Hyrax OR Haas OR “rapid maxillary expansion”):ti,ab OR (“maxillary transverse deficiency” OR “maxillary transverse hypoplasia” OR “maxillary transverse discrepancy” OR “posterior crossbite”):ti,ab)
